# Quantity - but not diversity - of secreted peptides and proteins increases with age in the tree frog *Pithecopus nordestinus*

**DOI:** 10.1590/1678-9199-JVATITD-2020-0105

**Published:** 2021-04-02

**Authors:** Douglas O. Mariano, Juliana M. Sciani, Marta M. Antoniazzi, Carlos Jared, Katia Conceição, Daniel C. Pimenta

**Affiliations:** 1 Laboratory of Biochemistry and Biophysics, Butantan Institute, São Paulo, SP, Brazil.; 2 São Francisco University, Bragança Paulista, São Paulo, SP, Brazil.; 3 Laboratory of Cell Biology, Butantan Institute, São Paulo, SP, Brazil.; 4 Laboratory of Peptide Biochemistry, Federal University of São Paulo (Unifesp), São José dos Campos, SP, Brazil.

**Keywords:** Peptides, Peptidomics, Proteins, Proteomics, Pithecopus nordestinus, Tree frog

## Abstract

**Background:**

Amphibians inhabit the terrestrial environment, a conquest achieved after several evolutionary steps, which were still insufficient to make them completely independent of the aquatic environment. These processes gave rise to many morphological and physiological changes, making their skin (and cutaneous secretion) rich in bioactive molecules. Among the tree frogs, the secretion is composed mainly of peptides; but alkaloids, proteins and steroids can also be found depending on the species. The most known class of biologically active molecules is the antimicrobial peptides (AMPs) that act against bacteria, fungi and protozoans. Although these molecules are well-studied among the hylids, AMPs ontogeny remains unknown. Therefore, we performed peptidomic and proteomic analyses of *Pithecopus nordestinus* (formerly *Phyllomedusa nordestina*) in order to evaluate the peptide content in post-metamorphosed juveniles and adult individuals.

**Methods:**

Cutaneous secretion of both life stages of individuals was obtained and analyzed by LC-MS/MS after reduction and alkylation of disulfide bonds or reduction, alkylation and hydrolysis by trypsin.

**Results:**

Differences in the TIC profile of juveniles and adults in both treatments were observed. Moreover, the proteomic data revealed known proteins and peptides, with slight differences in the composition, according to the life stage and the treatment. AMPs were identified, and bradykinin-potentiating peptides were observed in trypsin-treated samples, which suggests a protein source of such peptide (cryptide).

**Conclusion:**

In general, skin secretion contents were similar between juveniles and adults, varying in quantity, indicating that the different stages of life are reflected in the number of molecules and not on their diversity.

## Background

Amphibians were the first vertebrates to inhabit the terrestrial environment. This evolutionary step imposed on them a series of morphological and physiological changes [1, 2]. Most species begin as a free-swimming larva, breathing through gills. After undergoing metamorphosis, these animals start to live in the terrestrial environment, where they breathe through lungs and through the skin. However, these adaptations are insufficient to turn amphibians completely independent of the aquatic environment. For example, their fragile and gelatinous eggs depend, at least, on some humidity to develop [1, 2].

The anuran integument contains glands involved in different physiological process, such as cutaneous respiration, protection and thermoregulation [3-6]. Granular (poison) glands store a secretion involved in chemical defense [4, 7-10]. Once released, this secretion may be in contact with the mouth or eye mucosa of a predator and induce instantly symptoms such as ptyalism, hyperemic mucous membranes or nausea, avoiding predation [4, 11].

Granular secretion (or skin secretion) contains high number of biomolecules such as alkaloids, peptides, proteins or steroids and the toxin profile vary greatly among each taxonomic group or even among the species [4, 6-9]. Due to the high variability regarding the production of toxins, amphibians have been considered as natural apothecaries, capable of producing hundreds of compounds in their skin secretion. [12]

Most anuran tadpoles are aquatic and possess different phenotypes. For example, some researches show that when tadpoles are reared in the presence of a predator, they developed behavioral and morphological modifications [13-14]. These differences seem also to be reflected in terms of the molecular diversity in their skin secretin. Bókony et al. [15] studied the amount and natural variation of bufadienolides in *Bufo bufo* tadpoles. The authors observed an increase in toxin production when tadpoles coexist with more competitors. However, they did not find evidences linking a higher predation risk with an increase in chemical defenses in tadpoles.

Some researchers also evaluated the ontogenic toxin variation among bufonids. Hayes et al. [16] studied the qualitative and quantitative difference of several bufadienolides in *Rhinella marina* (*B. marinus*) during different life stages. The results revealed a higher diversity and quantity of bufadienolides in the eggs, which decreased during the tadpole phase and returned to rise after metamorphosis. In another study, Üveges et al. [17] found a rise in the quantity and variety of bufadienolides after hatchling stage in *B. bufo*. Furthermore, the authors also observed that tadpoles submitted to a limited food availability increased significantly the quantity of bufadienolides when compared to their *ad libitum* fed conspecifics.

Tree frogs are a widespread group among the anurans. Previously, tree frogs have been grouped into the Hylidae family; however, Duellman et al. [18] proposed a new phylogeny, classifying tree frogs in tree families: Hylidae, Phyllomedusidae and Pelodryadidae, all belonging to the unranked taxon Arboranae.

Adult tree frog skin secretion is one of the most studied among the anurans, being composed mainly by peptides. Several researches have already characterized biochemically and/or biologically these molecules, attributing to them diverse biological activities, such as antitumoral [19], immunomodulatory and insulinotropic [19-20], inflammatory [21] or vasoactive [22]. Besides that, these peptides have a prominent biological effect against microorganisms, such as antimicrobial [9, 23-24], antiparasitic [25], antiviral [19, 26], being known as antimicrobial peptides (AMPs).

Peptidome variation is a known phenomenon. Healthy individuals do present such idiosyncrasy [27] that can occur even daily [28]. Snakes have had their peptidomic variations assessed and demonstrated to be dependent, for instance, on gender. [29] Moreover, snake proteome seems to vary as well based on gender [30], post-translation modifications [31] or hunting needs. [32] Moreover, Roelants et al. [33] studied the origin and functional diversification of the amphibian AMPs. However, no research evaluating AMP distribution within the same species, comparing juveniles and adults has ever been performed, to the best of these authors knowledge.

**Figure 1.**
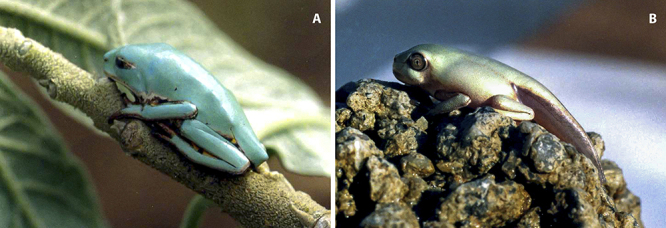
(A) Adult and (B) juvenile specimens of *Pithecopus nordestinus*.

Therefore, in order to evaluate the ontogenetic development of the AMPs and proteins secreted into the amphibian skin, we have performed the peptidomic and proteomic analyses of skin secretion of adult (Figure 1A) and juvenile (Figure 1B) specimens - under different sample preparation approaches - of *Pithecopus nordestinus* (formerly *Phyllomedusa nordestina*) and evaluated the peptide and protein content in post-metamorphosed juveniles and adult individuals.

## Methods

### Specimen collection

Specimens of *P. nordestinus* (family Phyllomedusidae) were collected in Angicos, Rio Grande do Norte State, Brazil during the rainy season, in February, 2004 (IBAMA 02001.005155/2008-13). They were brought to the Laboratory of Cell Biology of Butantan Institute, both in adult form (n = 15) and still in the form of eggs (3 spawning), that ecloded and developed until the form of complete metamorphosed juveniles (attested by the visual absence of tail). Adults were fed with crickets and cockroaches, and larvae were fed with flocked fish food until metamorphosis and life outside water, when newborn crickets and *Drosophyla* flies were then offered. All animal procedures were performed in accordance with the standards of the Ethics Committee on Animal Use of Butantan Institute (CEUAIB) (protocol #9532050216).

### Secretion attainment

In the laboratory, we collected the cutaneous secretion of adult specimens in the moment that they arrived from the field. Besides, we also collected the skin secretion from the 2-month young forms, when metamorphosis was completed. For the skin secretion collection each anuran was individually immersed in a Becker containing ultrapure water (same amount for each animal) and submitted to gentle mechanical stimulus with the tip of the fingers. Then, the secretions were pooled, according to their respective groups (adult or juvenile), lyophilized and kept in -80°C until we started the study.

### Reagents

All the employed reagents were purchased from Sigma Co. (St. Louis, MO, USA), unless otherwise stated.

### Sample preparation

Initially, the amount of protein in the skin secretion was determined using the bicinchoninic acid kit. Samples of 50 µg of proteins (two for each adult or juvenile skin secretion) were solubilized in in 100 µL of 8 M urea (diluted in 100 mM Tris-HCl, pH 8.5). Then, it was added 100 mM Tris(2-carboxyethyl)phosphine hydrochloride (TCEP) (dissolved in water, 5 mM final concentration) and incubated for 1h at room temperature; after that, 100 mM iodoacetamide (IAA) (dissolved in water, 10 mM final concentration) was added and the material was incubated for 1h, at room temperature and protected from light.

After the last step, one sample of 50 µg of protein (for each adult or juvenile skin secretion) was lyophilized and analyzed directly in the mass spectrometry, in order to identify natural peptides in the sample (termed non-digested sample). The other sample (50 µg for each adult or juvenile skin secretion) was diluted with 100 mM Tris-HCl pH 8.5 to reach urea concentration to 2 M and digested with 10 µL trypsin (Proteomics Grade, from porcine pancreas, Sigma Aldrich) (10 ng.µL^-1^ of protein) overnight, at 37 °C. The enzymatic reaction was stopped adding 50% acetonitrile (ACN) / 5% trifluoroacetic acid (TFA) and both materials were lyophilized to be analyzed in the mass spectrometry, in order to find proteins (termed enzymatically digested).

Prior to analyze in the mass spectrometer, both samples (enzymatically digested or non-digested) were desalted and concentrated using a ZipTip® C-18 pipette tips (Millipore, Massachusetts, USA), following the manufacture´s instruction, with final elution with acetonitrile containing 0.1% formic acid. The ZipTip® C-18 step was repeated twice and samples were pooled and dried by Speedvac to remove the solvent.

### Mass spectrometry (MS)

For the MS analysis, enzymatically digested or non-digested samples were dissolved in 10 µL of 0.1% formic acid (FA). Two microliter aliquots were inserted in a 15 cm x 50 µm Acclaim PepMap™ C-18 column (Thermo Scientific, Waltham, Massachusetts, USA) coupled to nano-chromatography EASY-nLC 1200 system (Thermo Scientific). The eluted content was automatically inserted in a Q Exactive Plus mass spectrometer (Thermo Scientific). Peptides were eluted in a linear gradient of 4-40% B1 (A1: 0.1% FA; B1: 80% ACN in 0.1% FA), at 300 nL/min during 100 min. Spray voltage was set at 2.5 kV and the mass spectrometer was operated in data dependent mode, in which one full MS scan was acquired in the m/z range of 300-1,500 followed by MS/MS acquisition using higher energy collision dissociation (HCD) of the ten most intense ions from the MS scan. MS and MS/MS spectra were acquired in the Orbitrap analyzer at 70,000 and 17,500 resolution (at 200 m/z), respectively. The maximum injection time and AGC target were set to 25 ms and 3.10^6^ for full MS, and 40 ms and 10^5^ for MS/MS. The minimum signal threshold to trigger fragmentation event, isolation window and normalized collision energy (NCE) were set to, respectively, 2,5.10^4^ cps, 1,4 m/z and 28. A dynamic peak exclusion was applied to avoid the same *m/z* of being selected for the next 30 seconds.

### Data processing

RAW files were directly loaded in the software Peaks Studio V7.0 (BSI, Canada) and the data was processed for *de novo* peptide sequencing and proteomic identification.[34] *De novo* peptide sequencing parameters were: MS and MS/MS error mass 10 ppm and 0.01 Da; methionine oxidation and carbamidomethylation as variable and fixed modification, respectively; when applied, trypsin was selected as the cleavage enzyme; average local confidence (ALC) ≥ 95%.

For proteomic identification the following parameters were adjusted: MS and MS/MS error mass were set to 10 ppm and 0.01 Da; methionine oxidation and carbamidomethylation as variable and fixed modification, respectively; when applied, trypsin was selected as the cleavage enzyme; maximum missed cleavages (3), maximum variable PTMs per peptide (3) and non-specific cleavage (both); the false discovery rate was adjusted to ≤ 0.5%; only proteins with the score ≥ 30 and containing at least 1 unique peptide were considered in this study. All data were analyzed against Phyllomedusinae (taxID: 192732) protein database (1271 entries) that was built by retrieving all UniProt entries associated with this subfamily, compiled on 05/18. Complementary analyses were also performed on the Amphibia (taxID: 8292) and the whole SwissProt databases.

### Protein analysis

Proteins identified in the proteomics analyses were grouped according to their gene ontology (GO) annotation, based on the described molecular function. The top ten peptides presenting ALC >95% that were not matched in the proteomic analyses, were individually BLASTed, limited to Phyllomedusinae (taxid: 192732).

## Results

**Figure 2.**
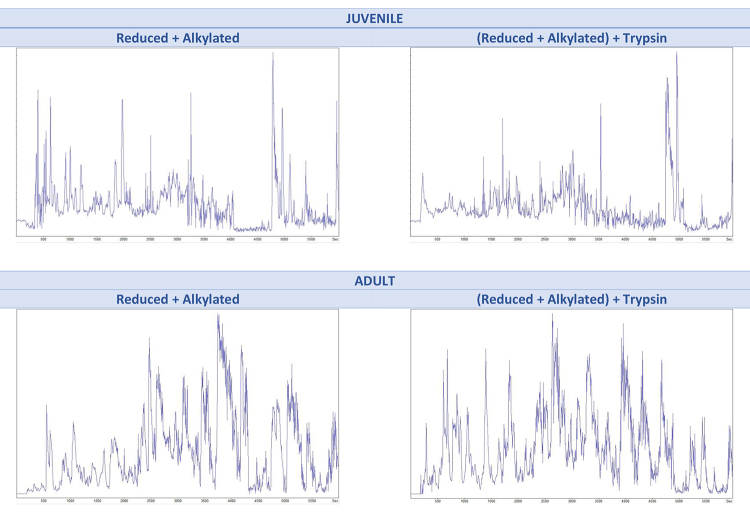
LC-MS/MS TIC chromatograms for the processed skin secretion of *Pithecopus nordestinus*, for comparison of the reduced/alkylated and the reduced/alkylated/digested samples of juvenile and adult individuals.

Figure 2 presents the total ion chromatogram (TIC) comparison of the reduced/alkylated (non-digested) and the reduced/alkylated/digested (enzymatically digested) samples for juvenile and adult individuals. It is possible to observe that the TIC chromatograms vary between juvenile and adults, in terms of number of peaks and peak group distribution. However, no significant variation observed between the experimental conditions for the same group, i.e., reduced/alkylated and reduced/alkylated/digested for juveniles are similar among themselves.

The proteomic data for each experimental condition were processed by Peaks Studio V 7.0, using the default parameters. The number of identified proteins, for each experimental condition was: 214/93 (total/redundancies removed) for reduced/alkylated juvenile; 376/200 (total/redundancies removed) for reduced/alkylated adult; 188/186 (total/redundancies removed) for reduced/alkylated/digested juvenile and 411/151 for reduced/alkylated/digested adult. These data are presented in Additional file 1.

**Figure 3.**
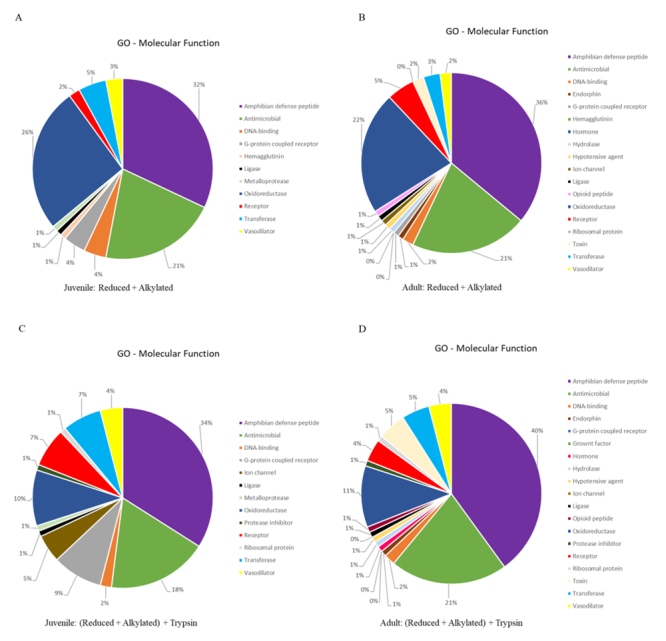
GO molecular function distribution of the identified proteins for *Pithecopus nordestinus*. **(A)** Reduced + alkylated from juveniles. **(B)** Reduced + alkylated from adults. **(C)** Reduced + alkylated + trypsin from juveniles. **(D)** Reduced + alkylated + trypsin from adults.

We extracted the protein identifiers from the results and analyzed them at the UniProt retrieve/ID mapping tool, according to the GO-Molecular Function (MF) identifier. The GO-MF results are present as pie chart, employing the same color-code, in Figure 3. It is noteworthy to mention that two of the three major classes of identified proteins present roughly the same percentage distribution (amphibian defense peptides: ± 35%; antimicrobial: ± 20%) in all analysis. However, the percentage of identified proteins classified as oxidoreductase decreased at half between juvenile/adult reduced + alkylated (± 24%) and juvenile/adult reduced + alkylated + trypsin (± 10%) treatments.

Some proteins classes are present only in adults, for both treatments (with or without digestion): endorphins, hypotensive agents, hydrolases, opioid peptides and hormones were not identified in juveniles, only in adults (Figure 3).

**Figure 4.**
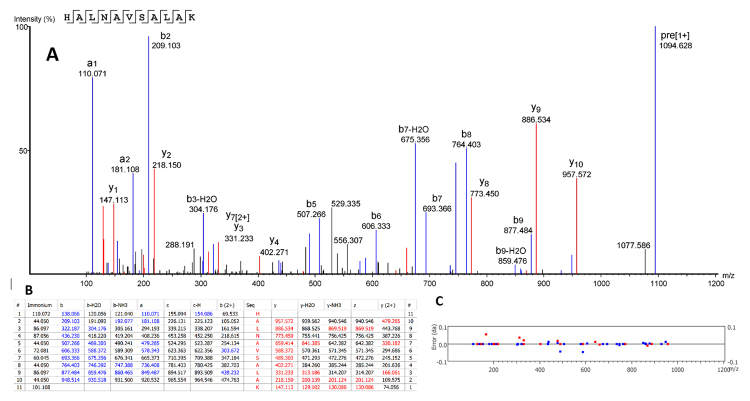
Representative Peaks Studio interpreted peptide sequencing. **(A)** MS/MS spectrum presenting the deduced sequence, as well as annotated daughter ions. **(B)** Ion peak list, with series b (blue) and series y (red) annotated. **(C)** Graph of error (Da) of each ion identified.

The top ten scored *de novo* sequenced peptides are presented in Table 1. The top 200 peptides are presented in Additional file 2, together with the full Peaks Studio report. One representative Peaks Studio interpreted MS/MS spectrum is presented in Figure 4A, together with the software quality control data, including matching ions (Fig. 4B) and mass errors (Fig. 4C). The depicted spectrum corresponds to a Phylloseptin fragment or analogue, identified for the juvenile individual (reduced/alkylated/digested) (HALNAVSALAK, Table 1).

**Table 1.** Top ten most significant (ALC > 95, minimum) *de novo* sequence peptides for the different experimental conditions and their respective BLAS hit, limited to Phyllomedusinae (taxid:192732)

	JUVENILE				ADULT			
	Reduced + Alkylated		(Reduced + Alkylated) + Trypsin		Reduced + Alkylated		(Reduced + Alkylated) + Trypsin	
	***De novo* peptide**	BLAST hit	***De novo* peptide**	BLAST hit	***De novo* peptide**	BLAST hit	***De novo* peptide**	BLAST hit
1	LGPALLTRP	*Hyposin-5*	RLLLPLR	*Sauvagine*	HDYFPK	*Leucine rich repeat containing 8 family, member D*	KADCVNFFWK	*Opioid receptor XOR1*
2	SPLRLS	*Bioactive peptide 1*	HALNAVSALAK	*Phylloseptin*	DHDYFPK	*Leucine rich repeat containing 8 family, member D*	DHDYFPK	*Leucine rich repeat containing 8 family, member D*
3	LLPLR	*Sauvagine*	YLDFVR	*FAT atypical cadherin 1*	MLQKNHMV	*Fibronectin leucine rich transmembrane protein 3*	VGKEAALAAAK	*Dermaseptin*
4	FWGTLAK	*Cullin-associated and neddylation-dissociated 1*	AAEDFLTLAK	*cullin-associated and neddylation-dissociated 1*	YLDFVR	*FAT atypical cadherin 1*	RPALLVR	*Hyposin*
5	LSLLPH	*Phylloseptin*	AFEELKELLQK	*sacsin*	RLPALLVR	*Balteatide*	LPTALNAVSALAK	*Phylloseptin*
6	WPPWQT	*Tryptophyllin*	LLGDTLSK	*Dermaseptin*	LLGDTLSKATN	*Dermaseptin*	HALNAVSALAK	*Phylloseptin*
7	FLSLLPH	*Phylloseptin*	HALNAVSALAK	*Phylloseptin*	WDPALDLKE	*Carbohydrate (keratan sulfate Gal-6) sulfotransferase 1*	RRPAFLRPK	*Hyposin*
8	NVSALAKWP	*Phylloseptin*	FDALFSLK	*NADH dehydrogenase subunit 4*	KNLLKYD	*Filamin A interacting protein 1*	AAGKGLWSTLK	*Dermaseptin*
9	KEAALAKNV	*Dermaseptin*	FLAFTK	*NADH dehydrogenase subunit 4*	AAGKGKEAALAAAK	*Dermaseptin*	AAAKAAGK	*Adenoregulin*
10	FLSLLPHA	*Phylloseptin*	TYQALLLAK	*NADH dehydrogenase subunit 4*	AAGKGLWSTLKQK	*Dermaseptin*	AAGKGKEAALAAAK	*Dermaseptin*

Table 1, as well as Additional file 2, present peptides that were exclusively *de novo* sequenced, regardless they were used or not for proteomic identification. It is possible to observe that for the juveniles, when only reduction and alkylation were performed, eight out of the ten presented peptides are indeed bioactive peptides. When trypsinization was performed on this sample, this figure drops to four peptides, indicating that proteins were also present in the undigested skin secretion. For the adults, these rates were 4:10 and 8:10, respectively. Nevertheless, the same phenomenon could be observed, e.g., the presence of proteins in the cutaneous secretion, as revealed by the enzymatic digestion. The full figures for the *de novo* sequenced peptides were: 3226 for juveniles reduced + alkylated, 4067 for juveniles (reduced + alkylated) + trypsin, 7533 for adults reduced + alkylated and 10046 for adults (reduced + alkylated) + trypsin. These values were obtained when processing the data using Peaks Studio default filters (data not shown).

**Figure 5:**
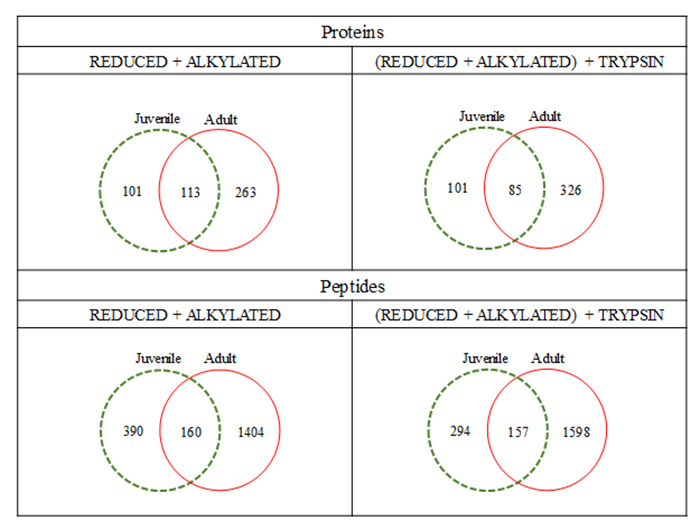
Venn diagrams displaying the overlapping protein and peptide identification of the contents of *Pithecopus nordestinus* cutaneous secretion, according to the employed experimental conditions.

We have compared the full datasets obtained after the proteomic processing in order to glimpse on the size of each proteome, regarding the individual’s age. Figure 5 presents the Venn diagrams for these comparisons. It is interesting to observe that, although no replicates were performed (we pooled the secretions), data are consistent: there are circa 100 overlapping identified proteins between juveniles and adults - regardless of sample processing - based on ~150 common peptides. Moreover, the unique protein and peptides were observed in comparison of juveniles and adults, for both methods, indicating that there are some molecules in common in their skin secretion, but on the other hand, several compounds (proteins and peptides) that are present only in adults or juveniles. This information is evident when peptides are being compared, when ~1000 are exclusive of adults and ~350 in juveniles, and ~150 in common.

Regarding the common proteins identified for juveniles and adults, the top 15 identifications (redundancies removed) are presented in Table 2. It is interesting to note that the reduced + alkylated condition yielded less ‘bioactive’ peptides than the (reduced + alkylated) + trypsin condition, in which all top-15 hits are ‘bioactive’ peptides. The first condition does present identified peptides, but - interestingly - only 5 among the fifteen hits are ‘bioactive’ peptides, the other 10 identifications correspond to physiological proteins. Upon analyses of the proteomic identification data, it is noteworthy to mention that the protein identification was not based on a single peptide, but several peptides covering all protein domains (data not shown).

**Table 2:** Top 15 common proteins (redundancies removed) between juvenile and adults, according to the methodological condition presented, sorted by the identification score.

Reduced + alkylated	(Reduced + alkylated) + trypsin
NADH-ubiquinone oxidoreductase chain 4 (EC 1.6.5.3)	Cruzioseptin-6 (CZS-6)
Cytochrome c oxidase subunit 1 (EC 1.9.3.1)	Dermaseptin-4
Dermaseptin sVI	Dermaseptin-1
Preprodermaseptin S10	[Thr6]-bradykinyl-Val,Asp (Bradykinin-related peptide RD-11)
Proteinase inhibitor PSKP-1	Dermaseptin PD-3-3
Bradykinin related peptide (Fragment)	Proteinase inhibitor PSKP-1
NADH-ubiquinone oxidoreductase chain 1 (EC 1.6.5.3) (Fragment)	Proteinase inhibitor PSKP-2
Cytochrome b (Fragment)	Bioactive peptide 1
Antimicrobial peptide	Phylloseptin-1 (PS-1)
Zinc fingers and homeoboxes 2	Phylloseptin-7 (PS-7)
Zinc finger E-box binding homeobox 1	Phylloseptin-12 (PS-12)
Titin	Dermaseptin-H2 (Dermaseptin-like peptide 2) (DMS2)
Sushi, von Willebrand factor type A, EGF and pentraxin domain containing 1	Phylloseptin-14 (PS-14)
Spen homolog, transcriptional regulator	Tryptophyllin-T3-2 (Pha-T3-2) (Tryptophyllin-10)
SLIT and NTRK-like family, member 1	Hyposin-HA5 (Hyposin-5)

## Discussion

The question of the how the Amphibian innate immune response changes along the development has already been assessed. Chen and Robert[35] have reviewed this subject and comments on the susceptibility of *Xenopus* adults and larvae to ranaviruses, being the latter much more susceptible to infection. Authors state that “AMPs may play an important role in inactivating viruses at their portals of entry and controlling infections prior to the onset of adaptive immune responses”. In another review, Romo et al. [36] present an overview of the innate immunity in vertebrates and analyze several components of this system, including: i) mucous secretion; ii) specialized skin; iii) cell mediate innate immunity; iv) proteins (enzymes, antibodies, cytokines) and v) antimicrobial peptides. Still, these authors comment that the humoral response components are poorly characterized in amphibians and reptiles, “making it difficult to understand the evolution of humoral innate immunity in vertebrates”. Nevertheless, the most important antimicrobial peptide for the immune innate response, the β-defensin, is present in vertebrates, such as fishes, lizards, birds, mammals and amphibians. [37]

On the other hand, it is known that the immune system of tadpoles changes right after the larvae metamorphosis. [38] Robert and Ohta [39] present an schematic overview of *Xenopus* immune system, and places the juveniles together with tadpoles, in terms of immune response, commenting that this would be a downregulated immune system, when compared to the larvae. Unfortunately, the authors do not evaluate the innate immune response in their work. Rollings et al. [40] also corroborate the metamorphosis impact on the cellular immune system.

So, in order to try to fill this information gap, we have biochemically assessed the peptide/protein contents of the cutaneous secretion of *P. nordestinus* individuals of different ages, specifically: juveniles (2-months old) and adults. Once the Phyllomedusinae has been already called ‘storehouses of bioactive peptides’ by Vittorio Erspamer [41], we chose the *omics* approach to study the subject, once a considerable large database would be available for datamining (currently, 1271 UniProt entries).

The proposed strategy was pretty straightforward: detect all peptides and proteins present in juvenile and adults and compare the identified molecules. For this, we prepared samples in two ways: i) reduced and alkylated (instead of crude) skin secretion; and ii) reduced, alkylated and trypsin-digested skin secretion.

The TIC chromatograms presented in Figure 2 show that there are already many peptides in the skin secretion, for the trypsin digestion did not significantly increase the number of peaks in the chromatogram (~3k to ~4k and ~7k to ~10k *de novo* peptides, respectively). Moreover, this figure also shows that the overall number of peptides is larger in adults, corroborating the first hypothesis of this work, i.e., the quantity of peptides changes along the ontogenetic development.

On the other hand, when analyzing Figure 3, it is possible to observe that, in spite of the increase in the number of peptides, the GO molecular function distribution (either digested or not) remains virtually the same, i.e., the diversity of the molecules present in the skin secretion does not change with ontogeny. The digested samples contain, as expected, an increased number of identified proteins, that contribute in the low % distribution.

Ujszegi et al. [42] performed an ontogenetic analysis of *Bufo bufo* (Bufonidae family), however, focusing on another class of molecules: steroids. The authors observed a decrease in steroid diversity in adult skin when compared to juvenile. On the other side, adults had a higher total quantity of steroids in the skin. In relation to the individual steroid quantity, each molecule showed an independent variation between the ontogenies.

Although we have performed a *shotgun* approach, i.e., the skin processed skin secretion solutions were directly analyzed without prior sample processing, we are confident in our results. Figure 4 presents a typical Peaks Studio processed MS/MS spectrum, in which it is possible to observe that: i) the daughter ions are representative of the whole sequence and are present in good relative intensity (panel A); ii) these ions provide enough information for deducing the sequence, particularly due to the presence of complete b and y series (panel B); and iii) the experimental error is very low (below 0,05 Da for daughter ions, panel C).

The quantity and diversity of the commonly identified proteins between juveniles and adults were also approached in this work. Figure 5 presents the Venn diagrams corresponding to these analyses. It is possible to observe that the, regardless of the sample processing, there is a ~1:1:3 proportion among exclusive juveniles:common:exclusive adults proteins based on a ~3:2:15 peptides proportion, for the above mentioned conditions. This means that adults present approximately twice the number of proteins than juveniles and four times the number of peptides; figures that, however, do not double the variety of proteins in adults.

Moreover, since the proportion of identified molecules remained the same in juveniles and adults, one cannot attribute the increase in the number of identified proteins to any specific molecular class, e.g., AMPs alone diversity have augmented. There seems to be an overall increase in the global proteome, in which all new peptides and proteins equally present in the adults’ skin.

Interestingly, there were several top-scored *de novo* sequenced peptides that are not classical AMPs (Dermaseptin, Phylloseptin, Tryptophyllin and Balteatide - Table 1), so the BIOPEP-UWM [43] database was queried, in search for cryptides. [44] Some of these peptides, particularly those derived from the leucine rich repeat containing 8 family, member D, matched this criterion. Their unique C-terminal FPK, a motif often found in ACE-inhibitors [45], makes it possible that these peptides would present such biological effect. The filamin A interacting protein 1 derived peptide (KNLLKYD) also matches another ACE inhibitor peptide (KRQKYDI), derived from porcine troponin. [46] Perhaps, the peptide derived from the opioid receptor XOR1, presenting a C-terminal FWK, could inhibit ACE as well, due to the similarity with the previous presented pattern. Peptide YLDFVR, matching FAT atypical cadherin 1, is similar to DFVAP, an ACE inhibitor from casein. [47] Nevertheless, complementary assays are necessary to support this hypothesis.

The fibronectin leucine rich transmembrane protein 3 derived peptide (MLQKNHMV) did not provide any evident match to any known bioactive peptide. However, the C-terminal - MV - is a known dipeptidyl peptidase IV inhibitor (DPP IV inhibitor), as well as the N-terminal; ML. [48]

Another sequenced peptide of particular interest was WDPALDLKE. This molecule displays features from two hazelnut peptides (DWDPK and AWDPE) that had been characterized as antioxidative peptides. [49]

Besides the obvious protective function of the AMPs identified in the skin secretion, we were able to sequence the C-terminal of ‘bioactive peptide 1’ (UniProt P84521), which complete sequence is <EQGEGGPYGGLSPLRFS (<E, pyroglutamic acid). This peptide might be considered a bradykinin potentiating peptide (BPP), due to its sequence features [50], but biological studies are still necessary to support this idea. Nevertheless, one actual BPP has already been isolated from *Phyllomedusa hypochondrialis* [51], so the current finding is not an isolated event. The short sequence LLPLR, also present in Table 1, matched ‘sauvagine’, which is a 40 amino acid peptide, also presenting a pyroglutamic acid at the N-terminal, which acts on diuresis, cardiovascular system and endocrine glands. [52] On the other hand, the short sequence LLPLR might be a species-specific variation of the C-terminal of bioactive peptide 1/BPP. However, complementary analyses are required.

Table 2 presents the data derived from the Venn diagrams in which the top-scored 15 proteins commonly identified between juveniles and adults are presented. It is noteworthy to mention that the protein profiles between the trypsin-digested and the undigested samples are profoundly different, namely: the digested sample presents only bioactive peptides (including two kazal-like protein inhibitors that display antibacterial activity but do not inhibit trypsin [53] whereas the undigested sample presents proteins.

The upraise in the number of identified peptides after trypsin digestion may have been caused by the fact that the bioactive peptides would be present in the form of (pre)pro-peptides and trypsin would have processed the peptides, making them detectable to the mass spectrometer, whereas when the sample is undigested, there would be peptides naturally present in the skin secretion, either as cryptides or cellular debris [54] that, when interpreted by Peaks Studio, derive from those proteins. Additional file 3 presents the peptides that were identified as belonging to NADH-ubiquinone oxidoreductase chain 4 (EC 1.6.5.3), the top scored common protein identified between juveniles and adults. It is possible to observe that protein identification was not based on few peptides, nor was it restricted to a specific domain of protein. The possible role of these peptides as cryptides still need to be evaluated. On the other hand, the presence of peptides derived from the constitutive cellular metabolism in the skin secretion have already been described [54].

## Conclusion

The current study has demonstrated that, for *P. nordestinus*, the ontogenetic development of the innate immune response (through AMPs) follows the direction towards the increase in the quantity of the secreted molecules, and not towards diversity. Moreover, analyses of the peptidome revealed that there might be new class of bioactive peptides (cryptides) displaying ACE inhibitory activity. These peptides would act in conjunction with the kinins present in the skin, potentiating smooth muscle effects that would serve as an anti-predation mechanism for both juveniles and adults.

## Supplementary material

The following online material is available for this article:

Additional file 1. Proteomically identified proteins and peptides for adults and juveniles, in both experimental conditions.

Additional file 2. Two hundred top-scored *de novo* sequenced peptides, together with the full Peaks Studio report.

Additional file 3. Proteomic identification of NADH-ubiquinone oxidoreductase chain 4 (EC 1.6.5.3) in the skin secretion of *Pithecopus nordestinus*. (A) Identification of the protein after sample reduction and alkylation. (B) Identification of the protein after sample reduction, alkylation and trypsin digestion. Blue bars: peptides identified by the proteomic approach. Gray bars: *de novo* sequenced peptides that aligned to the protein, as performed locally by Peaks Studio.
