# Supplementary material for: Quantity - but not diversity - of secreted peptides and proteins increases with age in the tree frog Pithecopus nordestinus
Source: J Venom Anim Toxins Incl Trop Dis. 2021 Apr 2;27:e20200105. doi: 10.1590/1678-9199-JVATITD-2020-0105 (PMC8020714; doi:10.1590/1678-9199-JVATITD-2020-0105)

J Venom Anim Toxins incl Trop Dis

https://doi.org/10.1590/1678-9199-JVATITD-2020-0105

Supplementary Material to "Quantity – but not diversity – of secreted peptides and proteins increases with age in the tree frog *Pithecopus nordestinus*"

Additional file 3. Proteomic identification of NADH-ubiquinone oxidoreductase chain 4 (EC 1.6.5.3) in the skin secretion of *Pithecopus nordestinus*. **(A)** Identification of the protein after sample reduction and alkylation. **(B)** Identification of the protein after sample reduction, alkylation and trypsin digestion. Blue bars: peptides identified by the proteomic approach. Gray bars: *de novo* sequenced peptides that aligned to the protein, as performed locally by Peaks Studio.

A)

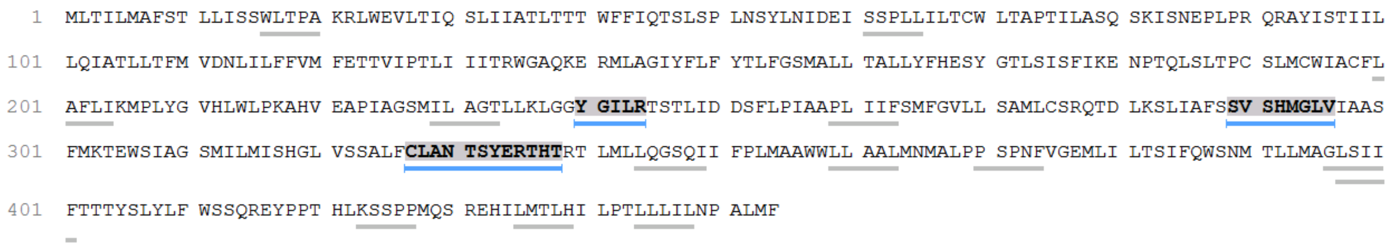

B)

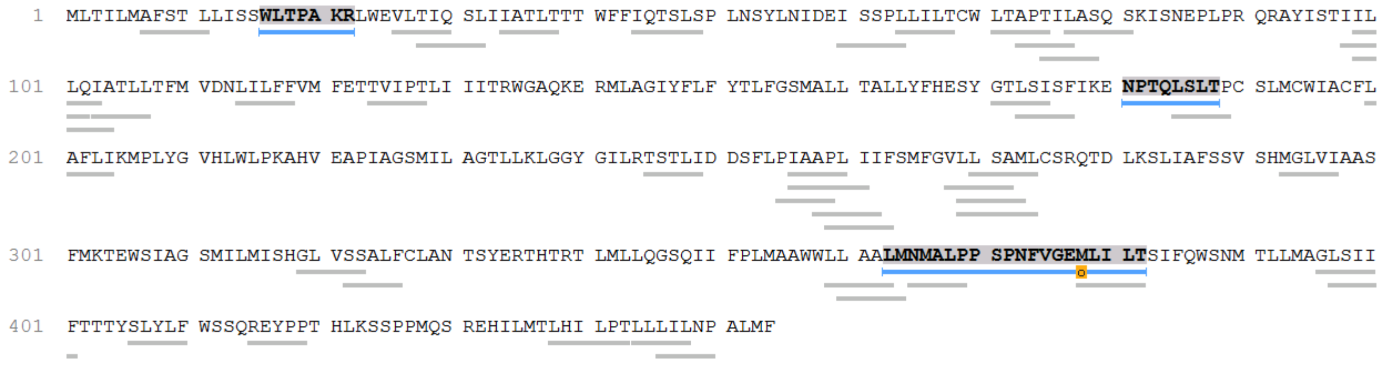

Supplement: Additional file 3. [file 1678-9199-jvatitd-27-e20200105-s3.pdf]
